# The Efficacy of Clinical Tests to Diagnose Evaporative Dry Eye Disease Related to Meibomian Gland Dysfunction

**DOI:** 10.1155/2022/3889474

**Published:** 2022-02-10

**Authors:** Jerry R. Paugh, Tiffany Nguyen, Alan Sasai, Elaine Chen, Melinda Thomas De Jesus, Justin Kwan, Andrew Loc Nguyen, Marjan Farid, Sumit Garg, James V. Jester

**Affiliations:** ^1^Southern California College of Optometry, Marshall B. Ketchum University, Fullerton, CA, USA; ^2^Department of Mathematics, California State University, Fullerton, CA, USA; ^3^Gavin Herbert Eye Institute, University of California, Irvine, CA, USA; ^4^Biomedical Engineering, University of California, Irvine, CA, USA

## Abstract

**Objectives:**

To determine the efficacy of widely available subtype clinical tests to characterize evaporative dry eye disease (EDED) related to meibomian gland dysfunction (MGD) compared to normal and to validate those clinical cut points in an independent sample.

**Methods:**

A diagnostic accuracy study (52 subjects), an investigator-masked study, was followed by a larger independent sample (364 subjects) analysis to confirm efficacy in normal and EDED subjects. All subjects were 18 years of age and older and were classified using a battery of clinical tests for dry eye that included symptoms, tear meniscus height, tear stability, ocular staining, evaporative-specific tests, and the Schirmer I test.

**Results:**

Normal (nondry eye; *n* = 26) and EDED (*n* = 26) subjects completed the efficacy study. The global tests of tear breakup time, staining, and symptoms all produced AUCs ≥ 0.70, representing acceptable discrimination. EDED-specific tests of eyelid marginal signs, gland secretion quality, and gland loss did not demonstrate acceptable test efficacy or differences between normal and EDED subjects. In a larger, independent sample of normal and EDED subjects, gland secretion quality and eyelid marginal score achieved acceptable diagnostic levels: AUCs of 0.789 (CI: 0.734–0.844) and 0.729 (CI: 0.648–0.810), respectively, but not lipid interferometry grade or lower eyelid gland dropout estimated using meiboscopy.

**Conclusions:**

Meibomian gland secretion quality is an efficient and useful functional indicator in EDED and should be incorporated into core outcome sets for this dry eye subtype.

## 1. Introduction

Dry eye disease (DED) is a common clinical condition, affecting 5 to 50% of the population, depending on the sampling approach and diagnostic criteria [1]. Of all the dry eye diseases, evaporative dry eye disease (EDED) related to meibomian gland dysfunction (MGD) appears to be the more prevalent subtype [2]. The prevalence of EDED may be much greater in Asian populations compared to other ethnicities [1, 3, 4].

As a common subtype of dry eye, EDED is a condition with major health and quality of life impacts that requires diagnostic methods that can also monitor treatment. In medical specialties, sets of clinical outcome criteria including those important to patients are developed by consensus groups into core outcome sets (COSs) [5]. COSs are used to standardize randomized clinical trials so that the effects of treatments can be uniformly assessed across trials such as in systematic reviews of treatment efficacy [5]. The parameters that comprise the COSs must be efficacious in diagnosing the medical condition and also quantitative to establish the severity of the condition and to monitor the response to treatment. Despite several consensus recommendations to diagnose EDED, [2, 6] tests have not been globally adopted that might comprise a COS battery for EDED related to MGD.

The 2011 MGD workshop report (diagnostic subcommittee [6]) suggested tests for EDED related to MGD diagnosis appropriate to a general clinic and additional tests for more specialized ocular surface clinics that may engage in clinical research. The tests for a general clinic included symptoms, lower tear meniscus height, tear osmolarity (if available), fluorescein breakup time, corneal and conjunctival staining, and the Schirmer I test. The general clinic recommendations also included observation of eyelid morphological features, gland expression/expressibility, and meibography. Recent surveys suggest that these general clinic tests are commonly used by ophthalmic practitioners [7, 8].

It was the purpose of this investigation to determine the test efficacy (sensitivity and specificity) of widely available clinical tests to characterize EDED related to MGD compared to normal and to validate those preliminary clinical cut points in an independent sample.

## 2. Methods

**Figure 1:**
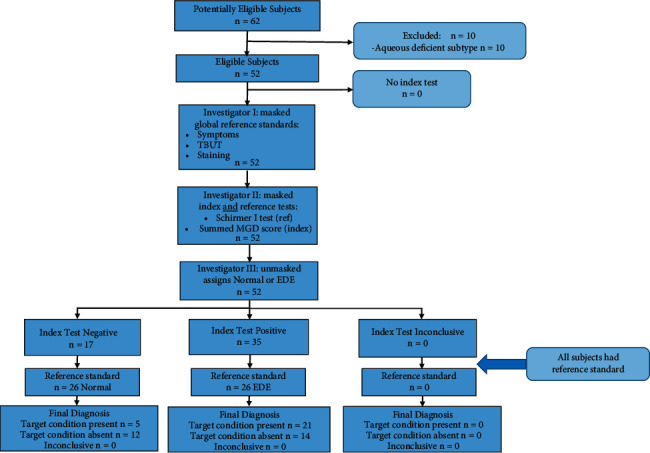
Participant flow diagram; STARD–2015 reporting guidelines.

This was a two-part investigation: an initial rigorous efficacy study (adhering to the STARD 2015 statement (Bossuyt et al. [9]), as shown in the flow diagram, Figure 1) and an independent sample conducted with identical methods and classification criteria. Both subject groups comprised a convenience sample of clinic-based subjects. Subjects were over the age of 18 years and provided written consent prior to the start of the study. These studies were approved by the Institutional Review Boards of Marshall B. Ketchum University and the University of California at Irvine.

Participant flow and investigator masking for diagnostic efficacy samples. The index test was the summed MGD score (as shown in the text) at a cut point of 5.4 on the 0–12 scale. The reference standard was clinical diagnosis of normal or EDE via the third masked examiner.

Major inclusion criteria were normal or dry eyes as determined by global DED tests, over age 18, and willingness to discontinue topical ocular drop use on the day of the assessment. Subjects were included if their dry eye management was stable for 30 days prior to enrollment. EDED was classified using either the lower eyelid gland secretion score ≥1.0 [10] or gland dropout using meiboscopy ≥1.0 [11]. Major exclusion criteria were blepharitis, ocular surgeries within 12 months of study start; active ocular allergy or infection; greater than mild ectropion, entropion, or ptosis; use of topical ocular medications except artificial tears; contact lens use; and punctal plugs within 30 days of study start. Aqueous deficient dry eye disease (ADDED) subjects were excluded based on the Schirmer I test for less than 5 mm of wetting in 5 minutes (without anesthesia) and tear meniscus height <0.20 mm [2].

Subjects for both studies underwent a comprehensive dry eye evaluation using the same tests from least to most invasive. For the efficacy study, separate masked examiners collected the global dry eye data (such as ocular history, tear stability, corneal, and conjunctival staining) or the specific meibomian gland data (such as lid marginal signs, gland expression, and meiboscopy). Symptoms (modified Schein, OSDI, and MGD-specific [12]), eyelid marginal signs (0 or 1 for present or absent orifice metaplasia, vascularity, capped glands, ridging, and marginal irregularity), fluorescein tear breakup time (TBUT; 2.0 *μ*l of 1.0% NaFl; yellow filter used, mean of three values), corneal (fluorescein) and conjunctival (lissamine green) staining with NEI and Oxford schemes, gland secretion (average score, entire lower eyelid, using the Bron 0–3 scale [10] using a cotton bud with gentle expression), lower eyelid meiboscopy (entire lower eyelid; percentage gland loss based on ½ and whole glands missing) [13], and the Schirmer I test without anesthesia were assessed. The central 8 glands of each eyelid were evaluated for meibomian glands yielding liquid secretion (MGYLS) using the Meibomian Gland Evaluator [14]. For the independent sample, the abovementioned tests were employed, but in addition, lipid layer appearance using white light interferometry (Yokoi scale, [15] custom apparatus, and 1–5 scale) was assessed.

The classification scheme used to assign subjects as normal or EDED was identical to that reported previously [16]. In brief, a subject was classified as EDED related to MGD if the OSDI was ≥13, the TBUT was <6.0 seconds [16], combined corneal and conjunctival staining >6.0 (NEI system, [17] 0–33 total scale; 6/33 ≈ 18% of total scale), and either meibomian gland secretion grade using 0.1 scale unit increments of >1.0 or gland dropout >1.0 [10]. Subjects were classified as normal (i.e., not dry eye) if TBUT was >6.0 seconds, total NEI staining was ≤6.0, and secretion and gland dropout scores were <1.0.

### 2.1. Statistical Methods

Statistical analysis was undertaken using Minitab version 18 (Minitab LLC, State College, PA, USA). An *a priori* sample size estimate was made to compare normal and EDED subjects relative to a cumulative score comprised of functional and morphological assessments (index test) [13]. The composite score was comprised of eyelid marginal changes (0–5), mean lower eyelid meibomian gland secretion grade (0–3), and gland dropout using meiboscopy (0–4). This provided a semicontinuous scale of 0–12.

Relative to a receiver operating characteristic (ROC) curve, an area under the curve (AUC) of 0.80 (excellent discrimination) vs. the chance level of 0.50 [18] was the efficacy target. Assuming Type I and II error levels of 0.05 and 0.20, respectively, equal standard deviations in the normal and EDED groups, and a two-tailed hypothesis, 13 subjects per group provided 0.81 statistical power.

For both the index test and the independent sample analyses, multiple regression analysis (continuous variables) and polytomous logistic regression (ordinal variables) were undertaken to examine the effects of age, sex, and dry eye subtype (normal vs. EDED) on the parameters of interest. Comparisons of normal vs. EDED were controlled for age and sex if significant from the continuous or ordinal analysis. For continuous data, ANOVAs including age, gender, and dry eye type were conducted, with Tukey pairwise tests for significant factors between normal and EDED subjects. The *p*values were two-tailed and adjusted for multiple comparisons with *p* < 0.05 considered significant. 95% confidence intervals were constructed for each comparison. For ordinal data, age, gender, and dry eye subtype were compared using odds ratios and 95% CIs; those CIs that included the null value of 1.0 were not considered significant.

The ROC curve analysis for AUC and optimum cut point (defined as maximal sensitivity and specificity, or the cut point with the greater sensitivity if these were unequal) was undertaken for the normal and EDED data.

## 3. Results

Twenty-six normal, 10 ADDED, and 26 EDED subjects completed the study. The mean ages (±SD) were 53.2 (±14.9), 55.7 (±6.9), and 61.0 (±17.1) for the normal, ADDE, and EDED subjects, respectively. No statistical difference was found for age in the three groups (ANOVA, *p*=0.184). The ADDE subject data were eliminated from the data set so that only normal and EDED subjects were compared.

AUCs, cut points, sensitivity, and specificity values were derived from ROC analysis (Table 1). The index measure, summed MGD score, attained 0.81 sensitivity, but only 0.46 specificity and an AUC of 0.578, slightly better than chance [18]. The summed MGD score did not differentiate normal from EDED subjects (*p*=0.960). The global dry eye tests of symptom questionnaires, TBUT, and staining scores achieved test sensitivities near or above 0.70, considered an acceptable level for an effective dry eye test [6], as opposed to the EDED-specific tests for a general clinic that did not achieve an acceptable level of discrimination.

Additional data were available from several prospective studies conducted concurrent with the efficacy study using identical test methods and classification criteria. The available data varied by test measure, up to a maximum number of 364 charts from clinically normal subjects and subjects having EDED. The EDED test data of this independent sample are summarized in Table 2 and were used for normal vs. EDED comparison and ROC curve and cut point determination.

Regression analysis was undertaken for marginal signs (0–5), gland secretion quality (0–3), and gland dropout (0–4), all from the lower eyelid of the right eye. This demonstrated a significant association with age for all three parameters (*p* values were <0.001 for marginal signs and secretion quality, and *p*=0.034 for gland dropout).

**Figure 2:**
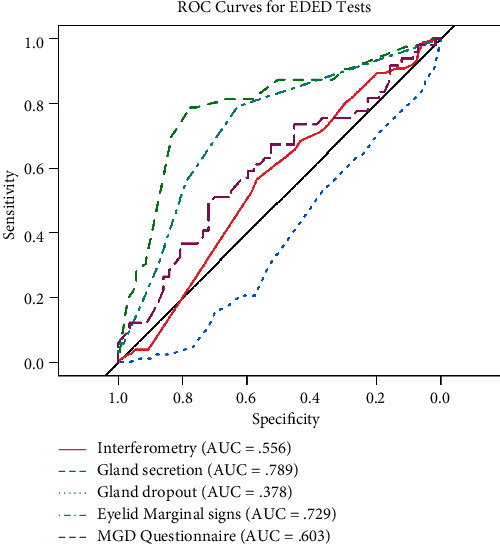
Receiver operating characteristic curves for EDE-specific tests, independent sample.

Receiver operating characteristic (ROC) analysis was undertaken for the five EDED-specific clinical tests. Comparisons between normal and EDED subjects are presented in Table 2, and the ROC curves in Figure 2.

Cut points were determined on the basis of maximal values sensitivity and specificity.

 
**Gland secretion:** AUC 0.789 (95% CI: 0.734–0.844), cut point 1.1 (0–3 scale; 0.1 unit scale increments); sensitivity = 0.79, specificity = 0.78; and *n* = 136 normal, 228 EDED subjects
 
**Marginal eyelid signs:** AUC 0.729 (95% CI: 0.648–0.810), cut point 4.5 (0–5 scale); sensitivity = 0.79, specificity = 0.63; and *n* = 94 normal, 93 EDED subjects
 
**MGD-specific questionnaire:** AUC 0.603 (95% CI: 0.494–0.713), cut point 40.5 (0–174 scale); sensitivity = 0.51, specificity = 0.71; and *n* = 57 normal, 88 EDED subjects
 
**Interferometry:** AUC 0.556 (95% CI: 0.484–0.629), cut point 2.1 (1–5 scale; 0.1 scale unit increments); sensitivity = 0.57, specificity = 0.57; and *n* = 85 normal, 190 EDED subjects
 
**Gland atrophy (meiboscopy):** AUC 0.378 (95% CI: 0.308–0.449), cut point 0.95 (0–4; continuous scale by percent of scale); sensitivity = 0.54, specificity = 0.36; and *n* = 100 normal, 229 EDED subjects

95% confidence intervals, diagnostic scale cut points, and subject sample sizes are summarized in text.

## 4. Discussion

It was the purpose of these investigations to determine whether routinely available clinical methods (such as the slit lamp biomicroscope, a cotton bud or Meibomian Gland Evaluator for gentle determination of gland secretion quality, and Finhoff transillumination for gland atrophy) can reliably identify EDED related to MGD by examination of functional and morphological changes in the lower eyelid. Under rigorous study design (such as double investigator masking), only the global clinical tests of symptom questionnaires, TBUT, and staining scores demonstrated adequate test efficacy (sensitivity and specificity >70%) [6] and the ability to distinguish between normal and EDED subjects (Table 1).

However, in the independent sample, gland secretion, using the Bron scale [10, 19] with 0.1 unit scale increments, demonstrated test efficacy by AUC (0.789) (Figure 2), sensitivity and specificity of 0.79 and 0.78, respectively, and statistically significant differentiation of normal vs. EDED subjects (Table 2). The cut point of 1.1 on a scale of 0–3 aligns with the Bron and coauthor's recommendation of deficient secretion at Grade 1 or greater [10]. Recently, Xiao et al. [20] also reported excellent discrimination for gland secretion (AUC of 0.98), on a 0–24 scale for 8 glands on the lower eyelid, although they did not propose a diagnostic cut point. Altered gland secretion is an indicator of adverse functional change, and this simple test appears useful in EDED diagnosis.

Eyelid marginal signs were not greatly different in normal and EDED subjects in the efficacy study (Table 1), and the AUC was not sufficiently diagnostic, possibly due to limited sample size (*n* = 26 normal and MGD subjects). In the independent sample data (text and Table 2), eyelid marginal signs did show adequate AUC and discrimination of normal vs. EDED subjects, but the cut point was too great, at 4.5 on a 0–5 scale, to provide an effective diagnostic test threshold. As well, marginal signs are associated with increasing age, so in our data, these summed marginal score changes do not seem useful for diagnosis and classification of EDED. Arita and coworkers [21, 22] previously reported high AUCs for eyelid marginal signs, which suggests they are a useful general, if not severity level sign in MGD.

Meibomian gland dropout of the entire lower eyelid via meiboscopy (0–4 scale, in percent) does not appear to be a viable indicator of EDED given the low AUCs in both samples (Tables 1 and 2 and Figure 2). This measure is a rough estimate of gland loss, and we conclude that imaging (i.e., meibography), which has much greater test efficacy [20–22], is necessary to characterize this important morphological change in EDED.

White-light tear interferometry of the tear lipid layer has been recommended by the DEWS II diagnostic subcommittee [2] to assist in subtyping dry eye as being of evaporative etiology. We examined lipid interference grade using the scale recommended by Yokoi and coworkers [15] (1–5 scale, with 0.1 scale unit increments) and found no difference between normal and EDED subjects (mean grades of 2.32 and 2.49, respectively) (Table 2) and an AUC equivalent to a coin flip. This result may have occurred due to the significant thickness range responsible for the colored lipid patterns of the Goto scale [23].

Relative to developing core outcome sets (COSs) for clinical trials in dry eye, it appears that a combination of global and specific tests for EDED is necessary. The present and recently reported data suggest that the global tests of symptoms, osmolarity, tear stability, and corneal and conjunctival staining are effective in establishing the diagnosis and severity of EDED. However, given the high test efficacy of both tear stability measurement and staining, it does not appear necessary to also include tear osmolarity as an additional global indicator of EDED. The cost of this test also mitigates against its widespread use. Assessment of gland secretion using the 0–3 scale [19] in 0.1 unit increments is a useful diagnostic indicator of meibomian gland function. Moreover, it represents a physiological measure due to the gentle pressure required to examine secretion opacity and viscosity. Meibomian gland functional assessment must be paired with morphological quantitation, which requires glandular imaging. With the exception of meibography, all of these tests plus the Schirmer I and tear meniscus height tests for aqueous production are suitable for a general clinic.

### 4.1. Study Limitations

These studies suffered from selection bias, or the use of the test under consideration to classify the condition, which tends to overestimate its efficacy [24]. Efforts should be made to characterize EDED with additional tests, keeping in mind the confounder of age, so that these and other clinical tests may be more accurately evaluated for efficacy.

In summary, it appears from the independent sample using clinical tests that only meibomian gland secretion quality is a useful specific indicator of the evaporative subtype of dry eye. Gland morphology assessed using meibography appears necessary to diagnose and quantitate changes associated with evaporative dry eye disease. Additional work should endeavor to more comprehensively assess the test efficacy of gland expressibility directly with the several available clinical methods and indirectly via the effect of gland patency on lipid layer thickness to establish cut points for diagnosis and treatment monitoring.

## Figures and Tables

**Table 1:** Efficacy study: test values and diagnostic parameters of EDED-specific and global dry eye tests.

Test parameter							
EDE-specific tests	Scale range	Normal *n* = 26 mean (SD or IQR)	EDED *n* = 26 mean (SD or IQR)	AUC (95% CI)	Cut point	Sensitivity (CI); specificity (CI)	Significance^a^ (95% CI)
Marginal signs	0–5	4 (3–5)	4 (3–5)	0.554 (0.40–0.71)	3.5	0.65 (0.47–0.83); 0.46 (0.27–0.65)	*p*=0.440; OR 0.64 (0.21–1.97)^b,c^
Glands expressing	0–8	4 (2–5; (*n* = 20)	2 (1–4; *n* = 23)	0.643 (0.48–0.81)	3.5	0.70 (0.52–0.88); 0.55 (0.36–0.74)	*p*=0.508; OR 0.66 (0.20–0.223)^b,c^
Gland secretion	0–3 in 0.1 unit steps	1.40 (0.6)	1.57 (0.6)	0.589 (0.44–0.74)	1.95	0.31 (0.13–0.49); 0.85 (0.71–0.99)	*p*=0.232*p* (−0.53–0.13)
Gland atrophy	0–4	1.34 (0.7)	1.57 (0.7)	0.612 (0.46–0.77)	1.25	0.69 (0.51–0.87); 0.58 (0.39–0.77)	*p*=0.590 (−0.54–0.31)
Summed MGD score	0–12	6.35 (2.0)	6.94 (2.0)	0.578 (0.42–0.74)	5.35	0.81 (0.66–0.96); 0.46 (0.27–0.65)	*p*=0.960 (−1.05–1.00)^**b**^
MGD-specific questionnaire	0–174	54.1 (37)	84.1 (32)	**0.739** (0.60–0.88)	80.5	0.65 (0.47–0.83); 0.75 (0.58–0.92)	*p*=0.021 (−44.8 to −3.8)

**Global tests**							
Modified Schein survey	0–24	7.7 (4.6)	11.2 (3.9)	**0.745** (0.61–0.88)	9.5	0.73 (0.56–0.90); 0.69 (0.51–0.87)	*p*=0.026 (−**5.48 to** −**0.37**)
OSDI	0–100	19.9 (16)	39.9 (23)	**0.757** (0.62–0.89)	34.4	0.65 (0.47–0.83); 0.85 (0.71–0.99)	*p*=0.008 (−27.68 to −4.28)
TBUT	Cont.	12.6 (9)	3.8 (1)	**0.916** (0.84–0.99)	5.95	0.92 (0.82–1.00); 0.80 (0.65–0.95)	*p* < 0.001 (4.10–11.50)
NEI staining	0–33	5.1 (4)	11.9 (7)	**0.822** (0.70–0.94)	7.50	0.77 (0.61–0.93); 0.81 (0.66–0.96)	*p* < 0.001 (−10.1 to −3.6)
Oxford staining	0–15	4.0 (2)	7.9 (3)	**0.838** (0.73–0.95)	6.5	0.73 (0.56–0.90); 0.92 (0.82–1.00)	*p* < 0.001 (−5.38 to −2.04)

^a^Normal vs. EDED; polytomous logistic regression (ordinal data) or multivariable regression analysis (continuous data; Tukey simultaneous tests, controlled for age and gender; *p*values adjusted for multiple comparisons). ^b^Significant for age (*p* < 0.05). ^c^Significant for sex (*p* < 0.05).

**Table 2:** Descriptive and inferential statistical data (OD data only); independent sample analysis.

Test parameter	Scale range	Normal median (IQR) or mean (SD)	EDED median (IQR) or mean (SD)	Significance^a^ (95% CI)
**Marginal Signs** Normal (*n* = 94) EDE (*n* = 93)	0–5	3 (2–4)	5 (3.25–5)	*p*=0.954; OR = 1.02 (CI = 0.54–1.91)^**b**^
**Gland Secretion** Normal (*n* = 136) EDE (*n* = 228)	0–3	1.0 (0.6)	1.6 (0.6)	*p* < 0.001 (−0.67 to −0.40)^**b,c**^
**Gland atrophy** (meiboscopy) Normal (*n* = 100) EDE (*n* = 229)	0–4	1.0 (0.6)	1.4 (0.8)	*p* < 0.001 (−0.56 to −0.18)^**b**^
**MGD specific Questionnaire** Normal (*n* = 57) EDE (*n* = 88)	0–174	55.1 (42)	69.4 (38)	*p*=0.285 (−20.22–5.99)^**c**^
**Interferometry** Normal (*n* = 85) EDE (*n* = 190)	1–5	2.32 (0.6)	2.49 (0.7)	*p*=0.176 (−0.29–0.05)^**b,c**^

^**a**^Normal vs. EDED; polytomous logistic regression (ordinal data) or multivariable regression analysis (continuous data; Tukey simultaneous tests, controlled for age and gender; *p*values adjusted for multiple comparisons). ^**b**^Significant for age (*p* < 0.05). ^**c**^Significant for sex (*p* < 0.05).

## Data Availability

Data supporting this research article are available from the corresponding author on reasonable request.
